# Supplementary material for: Aid when ‘there is nothing left to offer’: Experiences of palliative care and palliative care needs in humanitarian crises
Source: PLOS Glob Public Health. 2023 Feb 1;3(2):e0001306. doi: 10.1371/journal.pgph.0001306 (PMC10021221; doi:10.1371/journal.pgph.0001306)
Supplement: Attachment — Submitted filename: Response to reviewers PGPH-D-22-01377.pdf [file pgph.0001306.s001.pdf]

McMaster University

The Arnold Johnson Chair in Health Care Ethics

CRL Building, 2nd Floor
224-1280 Main Street West
Hamilton, Ont,
Canada L8S 4K1

Phone: 905.525.9140 Ext. 22879
Cell: 905.869.7082
Email: schwar@mcmaster.ca
www.humanitarianhealthethics.net

October 28, 2022

Response to reviewers - rebuttal letter - PGPH-D-22-01377 Aid when 'there is nothing left to offer': Experiences of palliative care and palliative care needs in humanitarian crises for *PLOS Global Public Health*

Dear Dr McCall,

Many thanks for your consideration of our paper. We very much appreciate the kind and helpful comments from the reviewers and have done our best to respond to them here.

Comments from the reviewers	Response from the authors
The paper is long, especially the results section. Some parts can be moved to an appendix, which I don't think is essential.	We acknowledge this comment and have shortened where possible but do not feel we can peel back more without losing something essential
I wonder to what extent the authors think that there is an imperative need for "a critical re-evaluation for humanitarian actors of what counts as success and failure in relation to death and dying, and to examine where and why palliative care is absent from the care they provide."<br><br>The reader may think that it's totally absent from the approach, both at HCW and system levels. If not true, you may wish to rephrase.	We strongly agree with the reviewer that this phrasing is misleading and have altered the sentence on pg 20, lines 435-438 as follows:<br><br>"Our findings suggest a critical re-evaluation for humanitarian actors of what counts as success and failure in relation to death and dying, and to examine where and why palliative care *can be even more present in the care they provide.*"
Apart investment, further research may be helpful. It looks like quantitative methods may be used, surveys, resource evaluation and post-implementation studies, among other. I suggest adding a perspective paragraph.	We agree with the reviewer and have added suggestions for pathways forward on pg 23, lines 500-504 as follows:<br><br>"Pathways forward for palliative care in complex humanitarian settings should include but not stop at time and resource investments. Apart from investment, further research would be helpful, including surveys, resource evaluation and post-implementation studies that would help quantify the impact of palliative care, and evaluate the benefits to patients, communities and care providers."

Our reference list has been reviewed and no updates were required.

Many thanks again for your interest and support of our submission.

Sincerely,

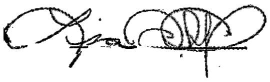

Lisa Schwartz, PhD, Professor, HEI
Arnold L Johnson Chair in Health Care Ethics
